# Low Dose Theophylline Showed an Inhibitory Effect on the Production of IL-6 and IL-8 in Primary Lung Fibroblast from Patients with COPD

**DOI:** 10.1155/2012/492901

**Published:** 2012-01-26

**Authors:** Jing Zhang, Ming-xiang Feng, Jie-ming Qu

**Affiliations:** ^1^Department of Pulmonary Medicine, Zhongshan Hospital, Shanghai Medical College, Fudan University, Shanghai 200032, China; ^2^Department of Thoracic Surgery, Zhongshan Hospital, Shanghai Medical College, Fudan University, Shanghai 200032, China; ^3^Department of Pulmonary Medicine, Huadong Hospital, Shanghai Medical College, Fudan University, Shanghai 200040, China

## Abstract

Chronic obstructive pulmonary disease (COPD) is characterized by the abnormal and chronic lung inflammation. We hypothesized that lung fibroblasts could contribute to the local inflammation and investigated whether low dose theophylline had a beneficial effect on fibroblasts inflammation. Subjects undergoing lobectomy for bronchial carcinoma were enrolled and divided into COPD and control groups according to spirometry. Primary human lung fibroblasts were cultured from peripheral lung tissue distant to tumor tissue. There was a significant increase in both the mRNA expressions and protein levels for IL-6 and IL-8 in fibroblasts in COPD group, and the values were negatively correlated with lung function (*P* < 0.05). For COPD fibroblasts, the protein levels of IL-6 and IL-8 decreased from 993.0 ± 738.9 pg/mL to 650.1 ± 421.9 pg/mL (*P* = 0.014) and from 703.1 ± 278.0 pg/mL to 492.0 ± 214.9 pg/mL (*P* = 0.001), respectively, with 5 **μ**g/mL theophylline treatment. In addition, theophylline at the dose of 5 **μ**g/mL reduced the increased production of IL-6 and IL-8 induced by 1 **μ**g/mL LPS in primary human lung fibroblasts. Our data suggest that lung fibroblasts participate in the chronic inflammation in COPD by releasing IL-6 and IL-8, and low dose theophylline can alleviate the proinflammatory mediators' production by fibroblasts.

## 1. Introduction

Chronic obstructive pulmonary disease (COPD) is one of the leading causes of morbidity and mortality worldwide and results in an economic and social burden that is both substantial and increasing [1]. It is currently the fifth leading cause of mortality worldwide and is predicted to become the third most common cause of death worldwide by 2030 [2]. COPD is characterized by the abnormal and chronic inflammation induced by cigarette smoking and other inflammatory insults in both small airway and lung parenchyma [3, 4]. 

Human lung fibroblasts are major structural cells orchestrating repair and remodeling in COPD. It is well acknowledged that the dysfunction of lung fibroblasts is partially responsible for small airway remodeling and peripheral emphysema in the progression of COPD [5, 6]. From another perspective, fibroblasts are gradually believed to be involved in the inflammatory process in the lung along with neutrophils, macrophages, T cells, and epithelial cells [7]. Data from recent studies have demonstrated that lung fibroblasts participate in the inflammation in response to diesel exhaust particles [8] and in asthma patients [9]. We therefore hypothesized that lung fibroblasts contribute to the chronic inflammation in the development of COPD. Our previous data showed that fibroblasts explanted from human distal lung tissue secreted higher levels of interleukin (IL)-6 and IL-8 in response to lipopolysaccharide (LPS)* in vitro*, while the productions of tumor necrosis factor (TNF)-*α*, IL-12p70, IL-1*β*, and IL-10 were at low levels or not altered [10]. We thus assumed that pulmonary fibroblasts in patients with COPD could participate in the local inflammation by producing a higher level of IL-6 and IL-8.

The control of the abnormal chronic local inflammation is regarded as a key strategy for managing COPD; however, with the relative glucocorticoids (GCs) insensitivity in COPD, there remain no effective anti-inflammatory treatments [11–13]. The alternative anti-inflammatory treatments which are currently available include low dose theophylline [14–16]. Theophylline is often described as a phosphodiesterase (PDE) inhibitor, but the concentrations that can achieve the anti-inflammatory effect are below the concentration at which it targets PDE and therefore avoid the side effect under high dose [17]. Clinical research in COPD showed that theophylline at the dose of 400 mg/day reduced both neutrophils counts and the levels of neutrophil elastase, myeloperoxidase, IL-8, and TNF-*α* in sputum [16, 17]. It is uncertain whether low dose theophylline would have a beneficial effect on fibroblasts inflammation. The purpose of this study was to address these issues in primary human lung fibroblasts explanted and cultured from subjects with COPD and non-COPD controls.

## 2. Methods

### 2.1. Subjects

Primary fibroblasts were cultured from peripheral lung tissue obtained at surgery for bronchial carcinoma. Patients were prospectively enrolled between October 2009 and February 2010. The demographic data including age, gender, smoking history, and past medical history were documented, and spirometry was performed preoperatively. Patients with clearly documented history of other lung diseases such as asthma, bronchiectasis, and interstitial lung disease were excluded. According to the classification of severity of COPD from Initiative for Chronic Obstructive Lung Disease (GOLD) 2009 [1], patients were divided into COPD group and control group (Table 1). The consecutive eight specimens from December 2009 to February 2010 were used for LPS treatment experiments. The study was approved by the Ethic Committee of Zhongshan Hospital of Fudan University where samples were collected, and it was registered at Chinese Clinical Trial Registry (no. ChiCTR-OCC-00000781). Written informed consent for the acquisition of material for research was obtained preoperatively.

### 2.2. Isolation and Cultures of Human Lung Fibroblasts

Human lung fibroblasts were isolated from surgical specimens of distant lesion-free and pleura-free lung tissue. Samples were immediately transferred into complete medium (Dulbecco's modified Eagle's medium (DMEM), 10% fetal calf serum (FCS), penicillin (100 ng/mL), and streptomycin (100 ng/mL)) (Invitrogen). The tissue was minced with a scalpel (1-2 mm^2^) and transferred into 25 cm^2^ culture dishes for primary culture at 37°C in 5% CO_2_. Cells were trypsinised when they were confluent and then passaged at a 1 : 3 ratio. Purified human lung fibroblasts cells of passage 3 were seeded at a density of 50 000 cells/well in 12 well plates and were grown in 1 mL complete medium for 24 h. Then cell-free supernatants were collected and total RNA was extracted for further experiments.

To confirm the purity of the cultured fibroblasts, the recovered cells were identified by their morphology, adherent nature, expression of vimentin (fibroblast marker) and lack of expression of cytokeratin (epithelial marker), vonWillebrand factor (endothelial markers), and SM22 (smooth muscle marker). 

### 2.3. ELISA

OptEIA ELISA kits for human interleukin (IL)-6 and IL-8 were purchased from BD. The level of protein of interest in cell-free culture supernatants was determined according to the manufacturer's instructions.

### 2.4. Real Time RT-PCR

Total RNA extraction was performed using Trizol Reagent (Invitrogen Life Technologies), and then 1 *μ*g of total RNA was transcribed using SuperScript ViLOTM cDNA synthesis kit (Invitrogen Life Technologies) according to the manufacturer's instructions.

Real-time PCR was performed using Express SyBR GreenERTM SuperMix with Premixed ROX (Invitrogen Life Technologies) following the manufacturer's instructions. The following primers were used: IL-6 forward, 5′- TAC CCC CAG GAG AAG ATT CC -3′; IL-6 reverse, 5′- GCC ATC TTT GGA AGG TTC AG -3′; IL-8 forward, 5′-TCT GCA GCT CTG TGT GAA GGT GCA GTT -3′; IL-8 reverse, 5′- AAC CCT CTG CAC CCA GTT TTC CT -3′; GAPDH forward, 5′-TGA GCA CCA GAT TGT CTC CT -3′; GAPDH reverse, 5′- GCA TCA AAG GTG GAA GAC TG -3′. PCR assays were performed in duplicate on the 7900HT real-time PCR machine (Applied Biosystems), and the cycler conditions were as follows: incubation for 2 min at 50°C followed by another incubation step at 95°C for 10 min, afterwards 15 s at 95°C and 1 min at 60°C for 40 cycles. Reaction specificity was evaluated by melting curve analysis which was performed by heating the plate from 55 to 95°C and measuring SYBR Green I dissociation from the amplicons. The calculation of threshold cycles (Ct values) and further analysis of these data were performed by the Sequence Detector software. The relative expression of HDAC2 mRNA in each sample was quantified and normalized to the GAPDH mRNA levels by the 2^-ddCt^ method [18]. 

### 2.5. *In Vitro* Treatments

Passage 3 cells were seeded as mentioned before and then cultured in complete medium with or without 5 *μ*g/mL theophylline for 48 h, followed by collecting supernatants and measuring IL-6 or IL-8. To evaluate the effect theophylline on the production of cytokines induced by LPS, purified human lung fibroblasts were grown in a complete medium (5% CO_2_ at 37°C) for 24 h. Subsequently, the human lung fibroblasts were treated with 1 *μ*g/mL LPS (Escherichia coli serotype 0111:B4, Sigma) for 48 h in the presence or absence of 5 *μ*g/mL theophylline, and then cell free supernatants were collected for later analysis.

### 2.6. Statistics

All statistical analysis was performed using GraphPadPrism 5.0 for Windows. D'Agostino and Pearson omnibus normality test was performed at first. If data were normally distributed, they were expressed as mean ± SD, and t-tests or repeated measures test of ANOVA followed by Dunnett's post-test were performed to calculate statistical differences between two groups or among three groups, respectively. Otherwise, data were expressed as median with interquartile range, and comparisons were made by Mann Whitney test between two groups. Correlation analysis was done by Spearman or Pearson test for normally or abnormally distributed data, respectively. Differences were considered significant at the level of  *P* < 0.05.

## 3. Results

### 3.1. Clinical and Demographic Features of the Subjects

Totally 18 subjects were enrolled into the current study and classified as COPD or control group according to GOLD 2009 [1]. The two groups were similar in age, gender, and smoking status (Table 1). The two groups differed significantly in lung function. As expected, the subjects in COPD group had lower FEV1% predicted and FEV1/FVC%, with values as 68.9 ± 18.7% versus 90.2 ± 5.5% (*P* = 0.013) and 57.4 ± 8.8% versus 79.3 ± 9.4% (*P* < 0.001), respectively.

### 3.2. A Significant Higher mRNA and Protein Level for IL-6 and IL-8 in Fibroblasts from Subjects with COPD

The primary cultured cells from human lung tissue appeared to have typical morphology of fibroblast, and immunostaining showed that >99% of the cells were vimentin positive. Staining with antibodies to cytokeratin, von Willebrand factor, and SM22 was negative, indicating that the cultures did not contain significant numbers of epithelial or mesothelial cells, endothelial cells, or smooth muscle cells.

**Figure 1:**
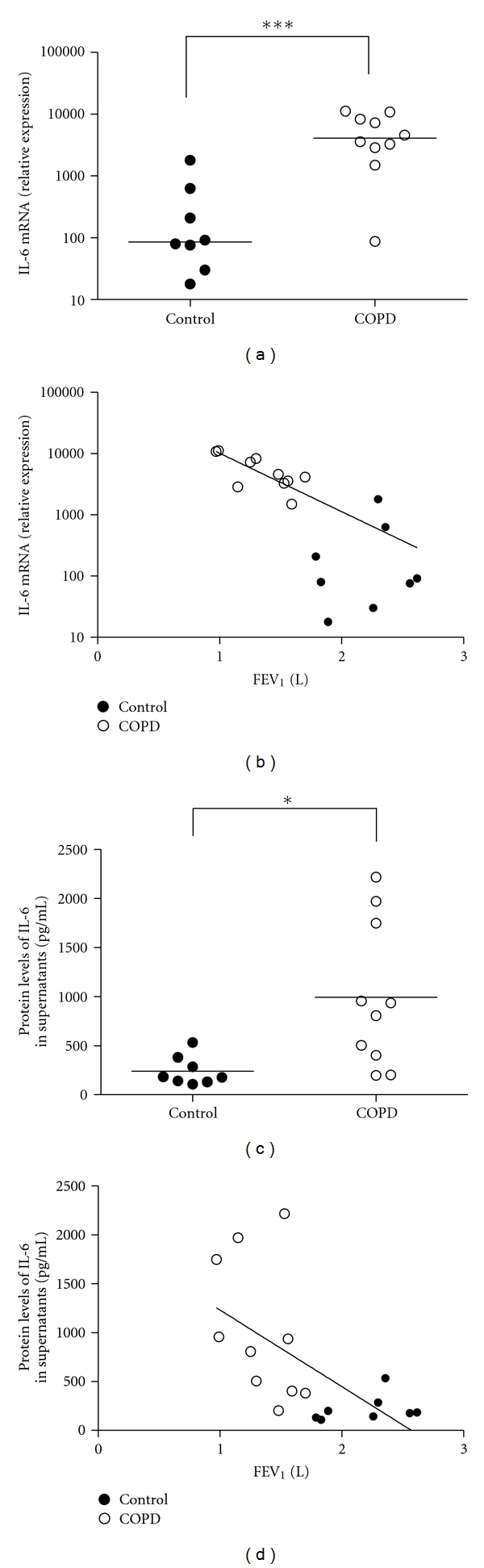
Increased mRNA expression and protein production of interleukin-6 (IL-6) in lung fibroblasts from COPD patients. (a) Real-time RT-PCR was adopted to evaluate the transcription levels of IL-6, and the relative expression was calculated based on the expression of GAPDH. ***P < 0.001, when compared to control group. (b) The relationship of IL-6 mRNA expression and FEV_1_. (c) The protein level of IL-6. ELISA was used to measure the concentration of IL-6 in the cell-free supernatants. *P < 0.05, when compared to control group. (d) The relationship of IL-6 secretion and FEV_1_.

**Figure 2:**
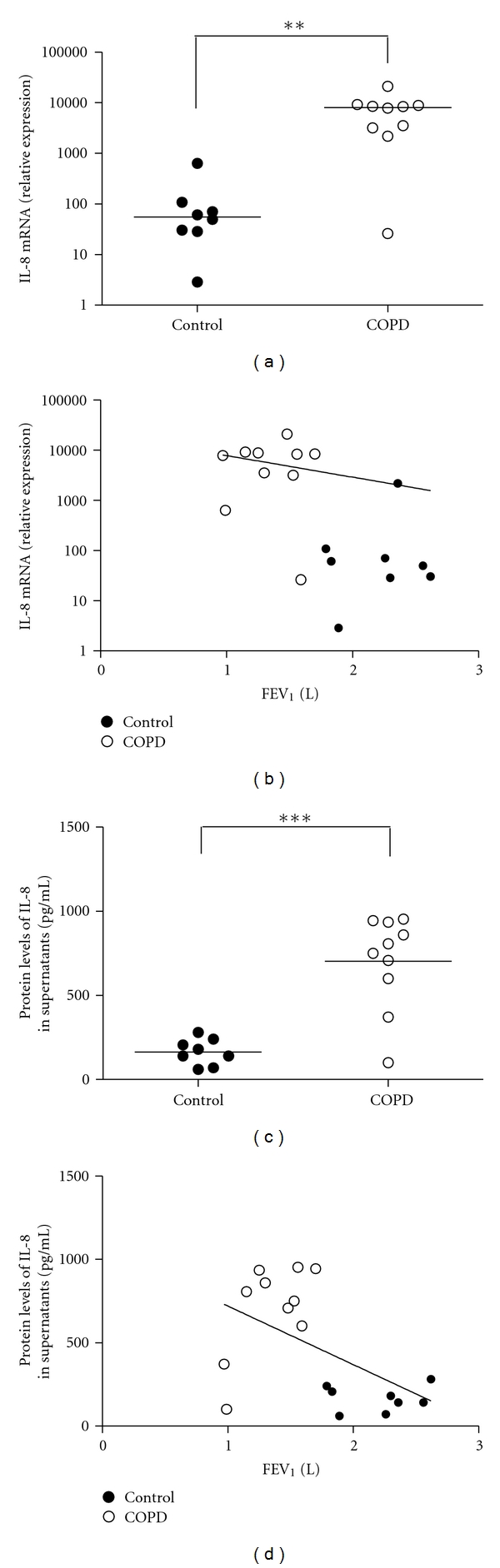
Increased mRNA and protein levels of interleukin-8 (IL-8) in lung fibroblasts from COPD patients. (a) Real-time RT-PCR was adopted to evaluate the transcription levels of IL-8, and the relative expression was calculated based on the expression of GAPDH. **P = 0.002, when compared to control group. (b) The relationship of IL-8 mRNA and FEV_1_. (c) The protein level of IL-8 of primary lung fibroblasts from subjects with COPD. ELISA was adopted to measure the concentration of IL-8 in the cell-free supernatants. ***P < 0.001, when compared to control group. (d) The relationship of IL-8 secretion.

There was a significant increase in the expression of mRNA for IL-6 (Figure 1(a)) and IL-8 (Figure 2(a)) for fibroblasts in COPD group. In addition protein levels were increased in the supernatants from the fibroblasts in COPD group compared with control group for IL-6 ((993.0 ± 738.9) pg/mL versus (241.5 ± 148.4) pg/mL, *P* = 0.012, Figure 1(c)) and for IL-8 ((703.1 ± 278.0) pg/mL versus (165.0 ± 77.5) pg/mL, *P* < 0.001, Figure 2(c)). As shown in Figures 1 and 2, the mRNA expression and protein production of IL-6 and IL-8 of fibroblasts from individual subject were negatively correlated with FEV_1_ (*P* < 0.05).

### 3.3. Low Dose Theophylline Inhibited IL-6 and IL-8 Production of Human Lung Fibroblasts Isolated from Subjects with COPD

**Figure 3:**
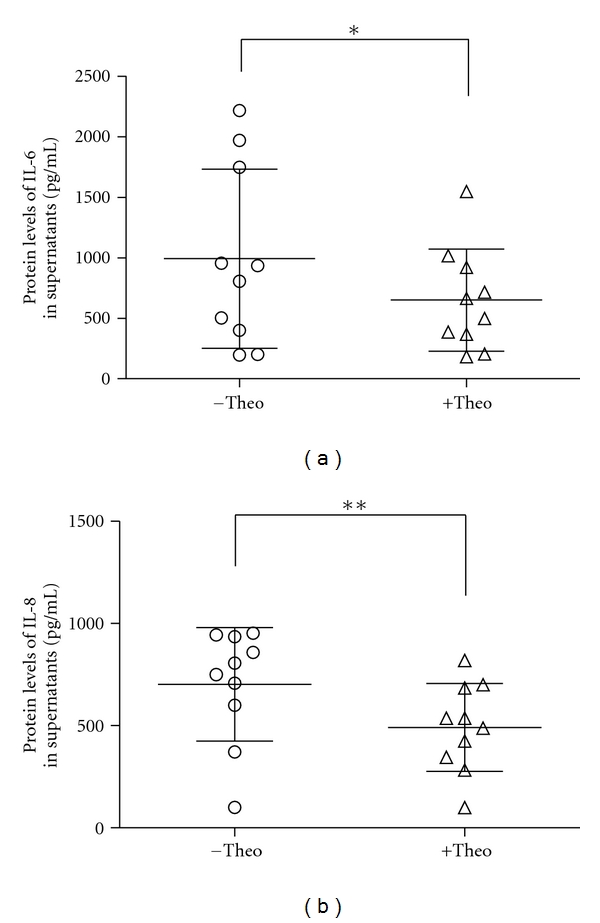
Low dose theophylline (5 *μ*g/mL) inhibited the production of interleukin-6/-8 in lung fibroblasts from COPD patients. Data were obtained from 10 independent cell lines. *P < 0.05, and **P < 0.01, when results between different treatments were compared. IL: interleukin; Theo: theophylline.

Theophylline at the concentration of 5 *μ*g/mL showed an inhibitory effect on the production of IL-6 and IL-8 in fibroblasts cultured from COPD patients. The levels of IL-6 and IL-8 decreased to (650.1 ± 421.9) pg/mL (*P* = 0.014, Figure 3(a)) and to (492.0 ± 214.9) pg/mL (*P* = 0.001, Figure 3(b)), respectively. No effect of theophylline on the cytokine production by control cells was observed. The treatment of theophylline had no effect on cell proliferation and viability (data not shown).

### 3.4. Low Dose Theophylline Partly Blocked the LPS-Induced IL-6 and IL-8 Secretion from Human Lung Fibroblasts

**Figure 4:**
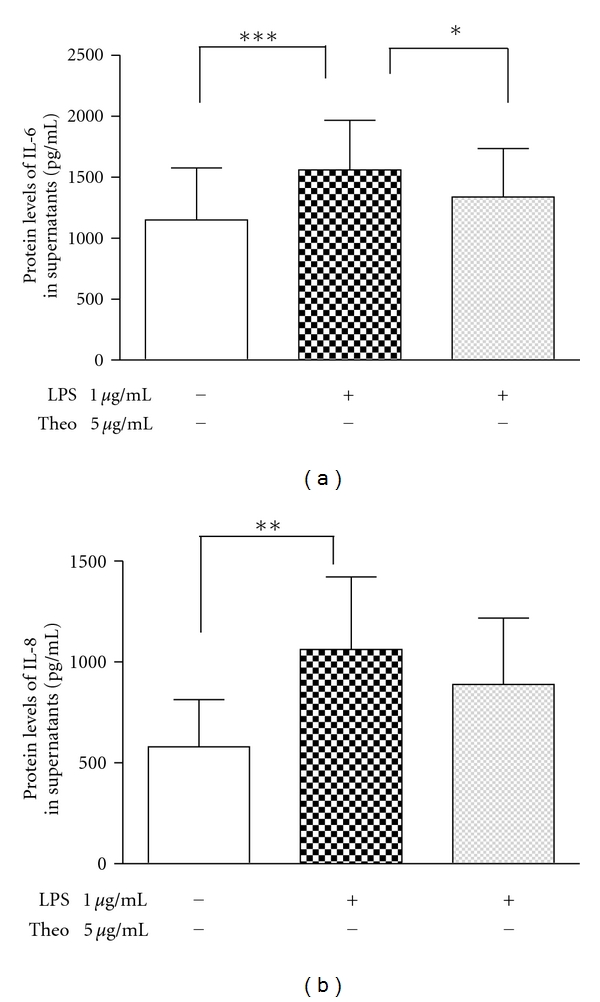
The effect of theophylline on the lipopolysaccharide-induced cytokine production in primary human lung fibroblasts. Data were obtained from 8 independent cell lines. The cells were cultured for 48 h at the absence or presence of 1 *μ*g/mL LPS, and the effect of theophylline at the concentration of 5 *μ*g/mL was analyzed. The production of IL-6 (a) and IL-8 (b) was quantitated by ELISA. The results were expressed as mean ± SD in picograms per milliliter. *P* < 0.05, when statistics were performed among −LPS/−Theo, +LPS/−Theo, and +LPS/+Theo. *P < 0.05, **P < 0.01, and ***P < 0.001, when results between two different treatments were compared. LPS: lipopolysaccharide; IL: interleukin; Theo: theophylline.

To evaluate whether LPS was affecting the production of IL-6 and IL-8 from primary human lung fibroblast, the cells were treated with or without 1 *μ*g/mL LPS. The dose of LPS was adopted based on our previous concentration-response experiments [10]. As shown in Figure 4, in response to 24-hour incubation of 1 *μ*g/mL LPS, the concentration of IL-6 in the supernatants of cultured fibroblasts was significantly increased from 1150 ± 426 pg/mL to 1559 ± 406 pg/mL (*P* < 0.05). A similar increase from 560 ± 205 pg/mL to 1087 ± 359 pg/mL was observed for IL-8 (*P* < 0.05). The treatment of theophylline at the dose of 5 *μ*g/mL partly blocked the significant increase induced by LPS.

## 4. Discussion

Chronic inflammation of the small airway and surrounding lung parenchyma involving a variety of cells and inflammatory mediators is presumably of major importance in the development and progression of COPD [3, 4]. Most of the previous studies have focused on the role of macrophages, neutrophils, and epithelial cells in the pathophysiology of the disease. In the current study, we found that compared with the control group with normal lung function, primary lung fibroblasts from subjects with COPD produced higher levels of IL-6 and IL-8 which were reduced by theophylline *in vitro*.

The increased levels of IL-6 or IL-8 we found in primary pulmonary fibroblasts from COPD patients were similar to those reported in sputum [19], exhaled breath condensate [20], and blood [21]. IL-6 is regarded to act as a proinflammatory cytokine and is also associated with epithelial apoptosis and injury in COPD [22]. IL-8 is a potent attractant for neutrophils and has been demonstrated to be responsible for the acute exacerbation and disease progression of COPD [19, 20]. Studies examining the circulation levels of IL-6 or IL-8 in COPD patients have suggested that inflammatory cells such as alveolar macrophages or epithelial cells are major sources of IL-6 or IL-8 [23–25]. However, Rao et al. [26] showed in a rat model that it is lung fibroblasts rather than alveolar macrophages that act as a significant source of IL-6. Ward et al. [9] showed that cultured myofibroblasts from asthma patients produced a higher level of IL-8 in the presence of IL-1. Recent research by He et al. [27] confirmed the expression of Toll-like receptors and its downstream signal transduction in lung fibroblasts, indicating that lung fibroblasts are also important target cells directly influenced by inflammatory stimuli. In the current study, we also observed the increased IL-6 and IL-8 production by primary human lung fibroblasts in response to LPS, a potent and ubiquitous proinflammatory agent. Taking these findings together, we can safely conclude that lung fibroblasts are at least one of the sources of IL-6 and IL-8 and contribute to the ongoing inflammation in COPD.

It is notable that theophylline at the dose of 5 *μ*g/mL reduced the *in vitro* IL-6 and IL-8 release by lung fibroblasts isolated from COPD patients or partly blocked the LPS-induced cytokines release. These results are consistent with the findings in clinical research and *in vitro* macrophage cultures [17, 28]. Iiboshi et al. [17] showed that four weeks of theophylline treatment reduced IL-8 level in sputum of stable COPD. Levels of IL-8 released by LPS-stimulated THP-1 cells and neutrophils were reduced by treatment with 10 *μ*g/mL theophylline [17]. A recent study also showed that low dose theophylline led to an additional reduction in sputum IL-8 and increase in total histone deacetylase (HDAC) activity in peripheral blood monocytes and neutrophils [28]. GCs are currently the most effective anti-inflammatory therapy for COPD; however, it has been confirmed that posttranslational modifications of glucocorticoid receptors (GRs), as well as decreased expression and activity of HDAC2, lead to the GCs resistance in patients with COPD [29]. Considering the relative GC-insensitivity in COPD, it is reasonable to use low dose theophylline as an available alternative or add-on anti-inflammatory therapy for COPD patients [14, 28, 29]. Importantly, lung fibroblasts are responsible for the extracellular matrix regeneration and maintenance, and GCs would interfere with cell proliferation and impair the tissue repair capacity of fibroblasts. Therefore, low dose theophylline might be more appropriate than GCs for inhibiting the inflammation in COPD fibroblasts. 

The exact mechanism of the anti-inflammatory effect of low-dose theophylline is not thoroughly known. It is assumed to be associated with the restoration of HDAC activity [30, 31]. HDAC suppresses inflammatory gene expression and appears to be the key regulator in the development and procession of COPD [32, 33]. The HDAC activity was found to be reduced in alveolar macrophage of COPD patients [34]. It was reported by Cosio et al. [30] that theophylline induced a six-fold increase in HDAC activity in COPD alveolar macrophage lysates and significantly enhanced dexamethasone suppression of induced IL-8 release. The mechanism underlying the inhibitory effect of theophylline on the cytokine release in COPD lung fibroblasts is required to be further investigated.

In summary, these data provided novel and direct evidences supporting the idea that lung fibroblasts participate in the abnormal inflammation in COPD. Low dose theophylline can reduce the release of proinflammatory mediators by fibroblasts, which would fortify the scientific rationale for its clinical use.

## Figures and Tables

**Table 1:** Clinical and demographic features of the subjects.

	COPD^†^	Control^‡^	*P* value
Gender (M/F)	5/5	5/3	NS
Age (yrs)	65.3 ± 9.8	69.5 ± 3.1	NS
Smoking history (pack-yrs)	43.0 ± 17.7	30.0 ± 10.0	NS
FEV1 (L)	1.35 ± 0.26	2.20 ± 0.33	<0.001
FEV1% pred	68.7 ± 18.7	90.2 ± 5.5	0.0128
FVC (L)	2.75 ± 0.76	2.97 ± 0.45	NS
FVC% pred	86.2 ± 20.5	89.0 ± 10.2	NS
FEV1/FVC%	57.4 ± 8.8	79.3 ± 9.4	<0.001

Data are presented as mean ± SD. COPD: chronic obstructive pulmonary disease; M: male; F: female; FEV1: forced expiratory volume in one second; % pred: % predicted; FVC: forced vital capacity; NS: nonsignificant. ^†^Subjects having an FEV1/FVC < 70%. ^‡^Subjects having an FEV1/FVC ≥ 70%.

## References

[1] Global Initiative for Chronic Obstructive Lung Disease Workshop report: global strategy for the diagnosis, management and prevention of chronic obstructive pulmonary disease. http://www.goldcopd.org/.

[2] World Health Organization WHO Statistical Information System (WHOSIS) http://www.who.int/whosis/whostat/2008/en/index.html.

[3] Chung KF, Adcock IM (2008). Multifaceted mechanisms in COPD: inflammation, immunity, and tissue repair and destruction. *European Respiratory Journal*.

[4] Hogg JC, Timens W (2009). The pathology of chronic obstructive pulmonary disease. *Annual Review of Pathology*.

[5] Sharafkhaneh A, Hanania NA, Kim V (2008). Pathogenesis of emphysema: from the bench to the bedside. *Proceedings of the American Thoracic Society*.

[6] Togo S, Holz O, Liu X (2008). Lung fibroblast repair functions in patients with chronic obstructive pulmonary disease are altered by multiple mechanisms. *American Journal of Respiratory and Critical Care Medicine*.

[7] Wupeng L, Zhang B, Cheng C, Yu-Keung M, Wong WSF (2008). Dendritic cell-derived interferon-*γ*-induced protein mediates tumor necrosis factor-*α* stimulation of human lung fibroblasts. *Proteomics*.

[8] Rao KMK, Ma JYC, Meighan T, Barger MW, Pack D, Vallyathan V (2005). Time course of gene expression of inflammatory mediators in rat lung after diesel exhaust particle exposure. *Environmental Health Perspectives*.

[9] Ward JE, Harris T, Bamford T (2008). Proliferation is not increased in airway myofibroblasts isolated from asthmatics. *European Respiratory Journal*.

[10] Zhang J, Wu L, Qu J-M (2011). Inhibited proliferation of human lung fibroblasts by LPS is through IL-6 and IL-8 release. *Cytokine*.

[11] Culpitt SV, Rogers DF, Shah P (2003). Impaired inhibition by dexamethasone of cytokine release by alveolar macrophages from patients with chronic obstructive pulmonary disease. *American Journal of Respiratory and Critical Care Medicine*.

[12] Barnes PJ, Adcock IM (2009). Glucocorticoid resistance in inflammatory diseases. *The Lancet*.

[13] Culpitt SV, Maziak W, Loukidis S, Nightingale JA, Matthews JL, Barnes PJ (1999). Effect of high dose inhaled steroid on cells, cytokines, and proteases in induced sputum in chronic obstructive pulmonary disease. *American Journal of Respiratory and Critical Care Medicine*.

[14] Barnes PJ (2005). Theophylline in chronic obstructive pulmonary disease: new horizons. *Proceedings of the American Thoracic Society*.

[15] Marwick J, Chung K (2010). Glucocorticoid insensitivity as a future target of therapy for chronic obstructive pulmonary disease. *International Journal of Chronic Obstructive Pulmonary Disease*.

[16] Kobayashi M, Nasuhara Y, Betsuyaku T (2004). Effect of low-dose theophylline on airway inflammation in COPD. *Respirology*.

[17] Iiboshi H, Ashitani J, Katoh S (2007). Long-term treatment with theophylline reduces neutrophils, interleukin-8 and tumor necrosis factor-*α* in the sputum of patients with chronic obstructive pulmonary disease. *Pulmonary Pharmacology and Therapeutics*.

[18] Fink L, Seeger W, Ermert L (1998). Real-time quantitative RT-PCR after laser-assisted cell picking. *Nature Medicine*.

[19] Keatings VM, Collins PD, Scott DM, Barnes PJ (1996). Differences in interleukin-8 and tumor necrosis facfor-*α* in induced sputum from patients with chronic obstructive pulmonary disease or asthma. *American Journal of Respiratory and Critical Care Medicine*.

[20] Ko FW, Leung TF, Wong GW (2009). Measurement of tumor necrosis factor-*α*, leukotriene B4, and interleukin 8 in the exhaled breath condensate in patients with acute exacerbations of chronic obstructive pulmonary disease. *International Journal of Chronic Obstructive Pulmonary Disease*.

[21] Lee TM, Lin MS, Chang NC (2008). Usefulness of C-reactive protein and interleukin-6 as predictors of outcomes in patients with chronic obstructive pulmonary disease receiving pravastatin. *American Journal of Cardiology*.

[22] Eddahibi S, Chaouat A, Tu L (2006). Interleukin-6 gene polymorphism confers susceptibility to pulmonary hypertension in chronic obstructive pulmonary disease. *Proceedings of the American Thoracic Society*.

[23] Barnes PJ (2004). Alveolar macrophages in chronic obstructive pulmonary disease (COPD). *Cellular and Molecular Biology*.

[24] Vecchio D, Arezzini B, Pecorelli A, Valacchi G, Martorana PA, Gardi C (2010). Reactivity of mouse alveolar macrophages to cigarette smoke is strain dependent. *American Journal of Physiology: Lung Cellular and Molecular Physiology*.

[25] Xu X, Wang H, Wang Z, Xiao W (2009). Plasminogen activator inhibitor-1 promotes inflammatory process induced by cigarette smoke extraction or lipopolysaccharides in alveolar epithelial cells. *Experimental Lung Research*.

[26] Rao KM, Ma JY, Meighan T, Barger MW, Pack D, Vallyathan V (2005). Time course of gene expression of inflammatory mediators in rat lung after diesel exhaust particle exposure. *Environmental Health Perspectives*.

[27] He Z, Zhu Y, Jiang H (2009). Toll-like receptor 4 mediates lipopolysaccharide-induced collagen secretion by phosphoinositide3-kinase-akt pathway in fibroblasts during acute lung injury. *Journal of Receptors and Signal Transduction*.

[28] Ford PA, Durham AL, Russell RE, Gordon F, Adcock IM, Barnes PJ (2010). Treatment effects of low-dose theophylline combined with an inhaled corticosteroid in COPD. *Chest*.

[29] Barnes PJ (2011). Glucocorticosteroids: current and future directions. *British Journal of Pharmacology*.

[30] Cosio BG, Tsaprouni L, Ito K, Jazrawi E, Adcock IM, Barnes PJ (2004). Theophylline restores histone deacetylase activity and steroid responses in COPD macrophages. *Journal of Experimental Medicine*.

[31] Ito K, Lim S, Caramori G (2002). A molecular mechanism of action of theophylline: induction of histone deacetylase activity to decrease inflammatory gene expression. *Proceedings of the National Academy of Sciences of the United States of America*.

[32] Ito K, Yamamura S, Essilfie-Quaye S (2006). Histone deacetylase 2-mediated deacetylation of the glucocorticoid receptor enables NF-*κ*B suppression. *Journal of Experimental Medicine*.

[33] Adcock IM, Tsaprouni L, Bhavsar P, Ito K (2007). Epigenetic regulation of airway inflammation. *Current Opinion in Immunology*.

[34] Ito K, Ito M, Elliott WM (2005). Decreased histone deacetylase activity in chronic obstructive pulmonary disease. *New England Journal of Medicine*.

